# The Fear of Contagion and the Attitude Toward the Restrictive Measures Imposed to Face COVID-19 in Italy: The Psychological Consequences Caused by the Pandemic One Year After It Began

**DOI:** 10.3389/fpsyg.2022.805706

**Published:** 2022-02-24

**Authors:** Nadia Rania, Ilaria Coppola

**Affiliations:** Department of Education Sciences, University of Genoa, Genoa, Italy

**Keywords:** COVID-19, Italy, psychological impact, fear of contagion, loneliness, mental health, social distance

## Abstract

The pandemic nature of COVID-19 has caused major changes in health, economy, and society globally. Albeit to a lesser extent, contingent access to shops and places to socialize the imposition of social distancing and the use of indoor masks is measures still in force today (more than a year after the start of the pandemic), with repercussions on economic, social, and psychological levels. The fear of contagion, in fact, has led us to be increasingly suspicious and to isolate ourselves from the remainder of the community. This has had repercussions on the perception of loneliness, with significant psychological consequences, such as the development of stress, anxiety, and, in extreme cases, depressive symptoms. Starting from these assumptions, this research was developed with the aim of deepening the perceptions that the participants have of their own mental health, loneliness, fear linked to contagion, and attitudes toward imposed social distancing. In particular, we wanted to analyze whether there is a relationship between perceived fear and the perceived level of mental health, loneliness, and attitude toward social distancing. Finally, we wanted to analyze whether there are differences related to gender, age, marital status, current working mode, and educational qualifications. The research, performed after the diffusion of the vaccination in Italy, lasted 14 days. The participants were 500 Italians who voluntarily joined the study and were recruited with random cascade sampling. The research followed a quantitative approach. The analyzed data, from participants residing throughout the national territory, allow us to return the picture of the perceptions that Italians have of the fear of contagion, of their level of mental health, of loneliness and of their attitude toward social distancing. In particular, the data show that fear of COVID-19 is an emotional state experienced by the entire population and that young people have suffered more from loneliness and have been less inclined to accept the imposed social distancing. The data that emerged should make policymakers reflect on the need to find functional strategies to combat COVID-19 or other health emergency crises whose effects do not affect the psychological wellbeing of the population.

## Introduction

The COVID-19 pandemic, which began in February 2020 in Italy, has had a dramatic impact on health, economic, and social levels. People have faced profound changes imposed by governments to reduce contagion, including the use of masks, social distancing, and more or less stringent lockdowns that have been repeated over the course of more than a year with different modalities and levels of restrictions. These changes have led to decidedly strong and invasive changes in everyday life with heavy repercussions on a psychological level In Italy, as of December 27, 2021, according to the data reported by the civil protection.^1^ The number of cases is equal to 5,647,313 and even if the number of recovered is significantly higher than number of dead; to this day, the number of infected is constantly increasing. The use of masks maintaining a distance of at least 1 m and vaccines is some of the measures taken by the government to counter the spread of the virus. The fear of contagion toward the most fragile people (those that are elderly or vulnerable), but also toward ourselves, has led us to be increasingly suspicious and to isolate ourselves from the remainder of the community for fear that others could be a danger to our safety. This has had repercussions on the perception of loneliness, felt particularly by the population in this period, leading to psychological consequences, such as the development of stress, anxiety (Porcelli, 2020; Tull et al., 2020; Rania and Coppola, 2021), and, in extreme cases, depressive symptoms. As stated by Briscese et al. (2020) in order to try to counter the spread of COVID-19, governments have applied measures of social distancing relying on the will of citizens to respect these restrictions. From the research conducted by the authors, it emerges that the expectations that people have about the expected duration of the lockdown influence their willingness to comply: if the restrictive measures are applied for a longer time than expected, their willingness to adhere to it will be less. In connection with the previous outbreak caused by the Syndrome (SARS), as noted by Wu et al. (2005), a risk factor for the development of depressive symptoms is the direct knowledge of people with SARS or having survived the disease. Finally, some authors have highlighted how the fear experienced during COVID-19 has had repercussions on people’s mental health, manifesting in feelings of anxiety, loneliness, uncertainty, and panic (Fitzpatrick et al., 2020).

### Fear of COVID-19 and Loneliness: Risk and Protection Factors

The COVID-19 pandemic has had repercussions, in the present, but it will also have them in the future, not only on people’s health, but also on different areas of life, including the economic and social sphere (Ceccato et al., 2021) with a significative level of traumatic stress, in women more than in men (La Rosa et al., 2021).

The fear experienced during the COVID-19 pandemic has had repercussions on psychological wellbeing: as noted, in fact, by Duong (2021), both fear and anxiety during the pandemic were determining factors in predicting forms of psychological distress, making emerging difficulties in mental wellbeing. Starting from the theory of attachment and the management of terror, referring to the pandemic situation we are going through, Steele (2020) suggests how the fear and anxiety experienced in one’s life are closely connected, in addition to the lack of coherent information, to fear of losing loved ones. Furthermore, Di Crosta et al. (2020) found that female gender, the perception of low economic stability and the fear of contagion are factors that negatively affect the psychological fallout due to COVID-19 and are predictors of a high symptoms of post-traumatic stress disorder. In the literature, it has been highlighted that infectious diseases are associated with fear, anxiety, and other psychological disorders (Cheng et al., 2004; Duong, 2021). COVID-19, as an infectious disease, can cause psychological distress, depression, anxiety, and fear (Lee and Crunk, 2020; Satici et al., 2020a; Duong, 2021). Research conducted by Di Crosta et al. (2021) shows how fear and anxiety about COVID-19 are predictors of changes in consumers, who under the effect of these emotions would feel the need to purchase goods necessary for survival. Fear, in particular, is defined as an emotional state, a response to a general malaise that is not well identifiable or quantifiable and clinically difficult to manage, particularly when it is linked to events of a broader nature, such as those related to terrorism and public health (Fitzpatrick et al., 2020). These cases include the COVID-19 pandemic, a direct threat that causes individual reactions. The speed and in-depth understanding of the exact methods of contagion have led people to feel panic and fear (Deniz, 2021), including the fear of being infected or infecting others, the risk of death, the loss of loved ones, and not receiving adequate care (Montemurro, 2020; Saricali et al., 2020; Satici et al., 2020b; Deniz, 2021). Several studies performed during the pandemic found that there was a progressive increase in COVID-19 fear around the world (Knipe et al., 2020) and that there is an association between fear and depression (Daly and Robinson, 2020; Lee et al., 2020; Lee and Crunk, 2020; Satici et al., 2020; Ye et al., 2020), which in severe cases can lead to suicide (Dsouza et al., 2020). Furthermore, during the COVID-19 pandemic, every society faced multiple challenges, including the pressure of social distancing and attention to contagion (Duong, 2021).

The most fragile population has been the most affected by COVID-19 and the effects caused by the restrictions imposed to limit its spread. Older people suffer most from the negative effects of COVID-19. Restrictive measures, fear, and loneliness have had negative repercussions on the resilience of people aged 65 and over, thus compromising their physical and psychological wellbeing (Plagg et al., 2020; Set, 2020; Savci et al., 2021). Esposito et al. (2021) underline how young participants due to the social restrictions imposed suffered of anxiety and depression; furthermore, Biviá-Roig et al. (2020) found that pregnant women during lockdowns suffered most from anxiety and depression. Rodríguez-Rey et al. (2020), moreover, underline how stress caused by COVID-19 is associated with alcohol use, more in women than in men.

Research conducted by Commodari and La Rosa (2020) highlights how young people have a lower perception of risk because they see COVID-19 as a less risky disease for them. However, previous research has shown that social isolation, regardless of age, is closely linked to symptoms of anxiety and depression (Matthews et al., 2019; Santini et al., 2020). Furthermore, during the pandemic, some authors found that the lockdowns caused mental illness even in the youngest (Lee et al., 2020; Coppola et al., 2021). Additionally, other studies (Porcelli, 2020; Tull et al., 2020) have found that being forced to stay at home has led to the development of greater stress, social isolation, loneliness, and anxiety about one’s health. Social distancing, in fact, one of the impositions dictated by many states in the hope of curbing the spread of the virus, has been defined as a possible factor that has contributed to the increase in dissatisfaction, anxiety (De Pedraza et al., 2020; Duong, 2021), and loneliness, with negative effects on wellbeing of the population (Boursier et al., 2020). Although social isolation and loneliness represent two distinct concepts, they are closely interrelated and are potential risk factors for suicide during and after the pandemic (Allan et al., 2021).

## Materials and Methods

The present research follows quantitative and exploratory methods. The questionnaire, administered online with the use of Microsoft Forms platform in open survey, was provided *via* a link sent by email, WhatsApp, discussion forums, and social networks, such as Facebook. The compilation of the protocol, *via* mobile phone or computer, took on average about 20 min per participant. No type of incentive was provided for the participants, who joined exclusively on a voluntary basis.

Before sharing the link, the researchers themselves filled out the questionnaire, in order to test its feasibility and functionality both through the use of smartphones and with a laptop and desktop pc. Both from the mobile phone and from the computer, the participant viewed four questions per page for a total of six pages. For each page, there was the possibility to go back to check or modify the answers given.

The convenience sample was recruited through random cascade sampling, starting from some subjects known to the research group, and involved participants who were at least 18 years old and Italian-speaking citizens.

The data were collected in accordance with the ethical recommendations of the Declaration of Helsinki and in compliance with the American Psychological Association (APA) standards for the treatment of human volunteers.

The questionnaire was proposed throughout Italy, thanks to its dissemination through the use of social media; however, most of the participants who filled out the questionnaire are from the same region as those who conducted the research. The research, of an exploratory nature, does not want to return a representative image of the Italian population but rather to give a picture of the perceptions of the population in relation to perceived fear and their own mental health (Lagomarsino et al., 2020).

Before starting the completion of the questionnaire, on an introductory page, the objectives of the study were described, the themes proposed, and an informed consent was offered to them through which the participants were asked to join voluntarily and they were informed that they could withdraw at any moment by closing the browser window. Only by accepting the consent could the participants start filling out the questionnaire. In addition, each participant was asked to build a code so that they could be contacted for further research. The code, therefore, allowed us to verify that the same participant has not filled out the proposed questionnaire several times.

The research was performed for 1 month and was carried out in April 2021; approximately, two-thirds of the questionnaires were compiled on the first 3 days of the questionnaire launch. Only fully completed questionnaires were analyzed.

### Measures

#### Fear of COVID-19 Scale

The FCV-19S (Ahorsu et al., 2020; Italian validation, Soraci et al., 2020) included seven items with a five-point Likert scale (from 1 = strongly disagree to 5 = strongly agree) that assess the fear of COVID-19. The higher the score, the greater the fear of COVID-19. The scale showed good internal consistency (*α* = 0.85).

#### Mental Health

The General Health Questionnaire with 12 items (GHQ-12) scale measures the state of mental health over the previous few weeks and was developed by Goldberg in the 1970s and validated in Italy by Piccinelli et al. (1993). The 12-item version, GHQ-12, is the most widely used (Elovanio et al., 2020). Participants had to report whether they experienced a particular symptom of mental distress according to a four-point Likert-type scale (“not at all,” “less than usual,” “more than usual,” or “rather more than usual”). The six positive items were corrected. Participants who answered “rather more than usual” or “more than usual” scored 1, while those who answered “less than usual” or “not at all” scored 0 (the so-called “0-0-1-1 method”; Elovanio et al., 2020). As pointed out by Piccinelli et al. (1993), this type of scoring, called conventional, “eliminated the problem of “middle and end users” and that of the “conceptual distance” between positions on the response scale. A total score ranged from 0 to 12 points; higher scores indicate worse health. The scale showed good internal consistency (*α* = 0.82).

#### Loneliness Scale

We used the Three-Item Loneliness Scale developed by Hughes et al. (2004) from the revised UCLA Loneliness Scale (Russell et al., 1980) in the Italian version of Solano and Coda (1994). It is a short scale for measuring loneliness in large surveys, and it assesses feelings of isolation, disconnectedness, and not belonging. Respondents are rated on a three-point Likert scale from 1 = hardly ever to 3 = often, with a total score ranging from 3 to 9 points; higher scores indicate greater loneliness. The three-item scale showed good internal consistency (*α* = 0.72).

#### Coronavirus Social Distance Attitudes Scale

The scale was composed of 14 items with eight expressing support to social distancing (*Positive Attitudes*, example item is “it is our duty as good citizens to follow social distance orders,”) and six expressing opposition to social distancing (*Negative Attitudes*, example item is “social distance orders violate my individual rights”; An et al., 2021). Items were answered using a five-point Likert scale (from 1 = strongly disagree to 5 = strongly agree). Both the positive social distance scale (*α* = 0.81) and negative social distance scale showed good internal consistency (*α* = 0.84).

The *demographic section* was composed of eight items exploring the demographic characteristics of the participants, their instruction level, and information about their work during the COVID-19 pandemic.

### Data Analysis

Descriptive statistics were calculated for sociodemographic characteristics, consisting of percentages, while the scores of Fear of COVID-19, General Health Questionnaire (GHQ-12), Loneliness, and Positive and Negative Social Distance Attitudes were expressed as means and standard deviations. To investigate the gender differences in relation to the constructs investigated, *t*-tests were used for independent samples. To compare the differences between our participants and the Italian normative sample and therefore in relation to the prepandemic data, *t*-tests were conducted for single samples. While variance analysis was used to investigate the differences between groups (age, marital status, current work mode, and educational qualification) in relation to the variables investigated, with *post-hoc* Tukey (for homogeneous variances) or Games-Howell (for non-homogeneous variances) between group comparisons in case of a significant overall *F*-value. Appropriate effect size statistics that adjust for differences in group sizes were obtained of Cohen’s d for *t*-tests and $\eta_{p}^{2}$ for ANOVAs. To explore the relationship between variables investigated, correlation analyses Pearson correlation coefficient was conducted. We used multiple linear step way regressions to calculate the univariate associations. SPSS (v. 20) software was used for these analyzes.

A *post-hoc* power analysis to evaluate power of this study was conducted using the software package, GPower (Faul and Erdfelder, 1992). The sample size of 500 was used for the statistical power analyses and a five predictor variable equation was used as a baseline. The recommended effect sizes used for this assessment were as follows: ssmall (*f*_2_ = 0.02), medium (*f*_2_ = 0.15), and large (*f*_2_ = 0.35; see Cohen, 1977). The alpha level used for this analysis was *p* < 0.05.

### Participants

A total of 500 adults from across Italy responded to the online questionnaire. Most respondents were women (86%), young adults (age *M* = 39.52 years, SD = 16.58; range 20–89), unmarried (47.7%), or married/cohabiting (44.7%), without children (62.4%), and with a secondary school diploma (41.9%).

The *post-hoc* analyses revealed the statistical power of this study was 0.67 for detecting a small effect, whereas the power exceeded 0.99 for the detection of a moderate to large effect size. Thus, there was more than adequate power (0.99) at the moderate to large effect size level but less than adequate statistical power at the small effect size level (Winnifred, 2009).

In Table 1, we report the sociodemographic characteristics in detail.

**Table 1:** Sociodemographic characteristics of the participants (*N* = 500).

Category variables	%
**Gender**	
Male	14
Female	86
**Marital status**	
Unmarried	47.7
Married/cohabiting	44.7
Separate/divorced	6.2
Widower	1.4
**Children**	
Participants with children	37.6
Participants without children	62.4
**Educational qualification**	
Junior high school	1.2
Secondary school	41.9
Graduation	39
postgraduate specialization	17.9
**Work arrangements during COVID-19**	
Unchanged	67.9
Smart working	26.4
Loss of job/work permit/leave	5.7
**Age**	
*M (SD)*	39.52 (16.58)
18–24	26.2
25–34	24.1
35–44	10.9
45–54	12.9
55–64	16.9
65 or older	9

## Results

### Fear of COVID-19

From the analysis of the results of the fear of COVID-19 scale (see Table 2) it emerged that there is a significant difference between the score obtained from the participants in the research and that reported by the normative sample. In fact, the participants in the research relating to fear of COVID-19 obtained a lower score compared to the normative sample, which refers to the first wave of the pandemic (Servidio et al., 2021). However, from the analysis of the *t*-test and ANOVA, with reference to the results obtained from the participants in the research, no significant differences emerged in the sociodemographic variables of gender, age, marital status, current work mode, and educational qualifications.

**Table 2:** Fear of COVID-19 scale: comparison between the average values of the participants and the average values of the Italian normative sample (Servidio et al., 2021).

	Fear of COVID-19		T (df)	*p*	Cohens’ *d*
	Participants	Italian normative sample during COVID-19			
	*M* (DS)	*M* (DS)			
Total sample	2.13 (0.75)	2.61 (0.87)	−14.274 (496)	0.000	0.59

### General Health Questionnaire

As seen from the data reported in Table 3, regarding the GHQ-12, the comparison with the normative sample shows a significant difference. The participants in the research reported higher levels of mental illness than the normative sample; moreover, when the comparison with the normative data is divided by gender, significant differences emerged. Both women and men reported a higher level of malaise than those in the female and male normative sample (Preti et al., 2007).

**Table 3:** Mental health comparison between the average values of the participants and the average values of the Italian normative sample pre-COVID-19 (Preti et al., 2007).

	GHQ-12		*t*(df)	*p*	Cohens’ *d*
	Participants	Italian normative sample			
	*M* (DS)	*M* (DS)			
Total sample	6.84 (3.04)	1.8 (2.3)	36.948 (496)	0.000	
Male	6.67 (2.9)	1.4 (2.0)	15.09 (68)	0.000	2.11
Female	6.89 (3.05)	2.5 (2.6)	29.6 (422)	0.000	1.55

However, no significant differences emerge from the comparison with the averages recorded during the first wave of the pandemic (Coppola et al., 2021, see Table 4). From the analysis of the results reported by the participants, there were no significant differences in relation to age, gender, or educational qualifications.

**Table 4:** Mental health comparison between the average values of the participants and the average values of the first wave (Coppola et al., 2021).

	GHQ-12	
	Participants	Italian sample during COVID-19
	*M* (DS)	*M* (DS)
Male	6.67 (2.9)	6.01 (3.07)
Female	6.89 (3.05)	6.45 (3.04)

Regarding marital status, however, a significant difference emerged between those who were single (*M* = 7.3, SD = 3.11) and those who were married/cohabiting (*M* = 6.45, SD = 2.98), *F*(3, 493) = 4.27, *p* < 01, $\eta_{p}^{2}$ = 0.03. The former report lower mental wellbeing than the latter. With regard to the current working mode, a significant difference emerged between those who reported an unchanged mode (*M* = 6.43, SD = 2.74) and those who were smart working (*M* = 6.35, SD = 3.27) or had lost their jobs (*M* = 8.71, SD = 2.71). Those who continued to work without changes reported lower mental wellbeing than those who were smart working, but higher mental wellbeing than those who lost their jobs, *F*(2, 293) = 5.11, *p* < 01, $\eta_{p}^{2}$ = 0.033.

### Loneliness

Regarding loneliness, significant differences emerged from the comparison with the normative sample (Caputo, 2017, see Table 5). The participants in the research reported a lower level of loneliness than the normative sample. Similarly, a significant difference emerged from the comparison with the normative sample divided by gender with regard to women. The participants in the research report a lower level of loneliness than the women in the normative sample. No significant differences emerged from the comparison between the male participants in the research and the males of the normative sample.

**Table 5:** Loneliness comparison between the average values of the participants and the average values of the Italian normative sample (Caputo, 2017).

	UCLA		*t*(df)	*p*	Cohens’ *d*
	Participants	Italian normative sample			
	*M* (DS)	*M* (DS)			
Total sample	5 (1.56)	5.46 (2.06)	−6.555 (496)	0.000	0.25
Male	4.8 (1.4)	4.94 (1.92)	−8.484 (68)	0.399	
Female	5.04 (1.58)	5.58 (2.08)	−7.092 (422)	0.000	0.29

Regarding the comparison between the averages recorded during the first wave of the pandemic and the data from the participants in the research regarding loneliness, significant differences emerged (Rania and Coppola, 2021, see Table 6). Both women and men reported a level of loneliness lower than the average recorded during the first wave of the pandemic. From the analysis of the results reported by the participants in the research, however, no differences emerged in relation to the current work mode and educational qualifications. However, differences emerged with respect to the age groups, particularly between those who were in the 18–24 age group (*M* = 5.46, SD = 1.56) and those who were in the 25–34 age group (*M* = 4.86, SD = 1.63), the 35–44 age group (*M* = 4.70, SD = 1.46), the 55–64 age group (*M* = 4.82, SD = 1.49), and those 65 or older (*M* = 4.44, SD = 1.18), *F*(5, 491) = 4.58, *p* < 0.01, $\eta_{p}^{2}$ = 0.05 Participants in the 18–24 age group reported a higher level of loneliness. Regarding marital status, the analysis of the results shows a significant difference between those who are single (*M* = 5.26, SD = 1.60), those who are married/cohabiting (*M* = 4.73, SD = 1.46), and widowers (*M* = 4, SD = 0.58). The former shows a higher level of loneliness than those who are married or widowed. Furthermore, a significant difference emerges between those who are divorced/separated (*M* = 5.23, SD = 1.76) and those who are widowers (*M* = 4, SD = 0.58). The former reports a higher level of loneliness than the latter, *F*(3, 493) = 4.27, *p* < 01, $\eta_{p}^{2}$ = 0.03.

**Table 6:** Loneliness comparison between the average values of the participants and the average values of the first wave (Rania and Coppola, 2021).

	UCLA		*t*(df)	*p*	Cohens’ *d*
	Participants	Italian normative sample during COVID-19			
	*M* (DS)	*M* (DS)			
Male	4.8 (1.4)	5.23 (1.71)	−2.570 (68)	0.012	0.28
Female	5.04 (1.58)	5.68 (1.97)	−8.394 (422)	0.000	0.36

### Positive Social Distance

Regarding the analysis of positive attitudes toward social distancing during COVID-19, a significant difference in relation to gender emerged from the comparison with the normative sample (An et al., 2021, see Table 7). The female participants in the research obtained a higher score than the women in the normative sample. Conversely, there were no significant differences between the scores of the male participants and the scores reported by the men in the normative sample. The analysis of the results reported by the participants in the research did not reveal any significant differences based on gender, marital status, current work modes, or educational qualifications. Instead, a significant difference emerged in relation to age, and in particular, between those who are in the age 25–34 group (*M* = 4.08, SD = 0.59) and those who are in the age 45–54 group (*M* = 3.66, SD = 0.83) and 65 or older (*M* = 4.36, SD = 0.46). The younger participants reported a positive attitude toward social distancing higher than those who are in the intermediate age group, but lower than older people. Furthermore, there was a significant difference between those who are in the age 35–44 group (*M* = 3.8, SD = 0.82) and the age 45–54 group (*M* = 3.66, SD = 0.83) and those who are in the age 55–64 group (*M* = 4.18, SD = 0.52) and 65 or older (*M* = 4.36, SD = 0.46), *F*(5, 491) = 9.81, *p* < 0.01, $\eta_{p}^{2}$ = 0.09. Older people report higher positive social distancing scores than younger people.

**Table 7:** Positive attitudes toward comparison between the average values of the participants and the average values of the Italian normative sample (An et al., 2021).

	Positive social distance		*t*(df)	*p*	Cohens’ *d*
	Participants	Italian normative sample during COVID-19			
	*M* (DS)	*M* (DS)			
Male	3.84 (0.89)	3.73 (0.97)	NS	0.303	
Female	4.04 (0.59)	3.94 (0.91)	3.624 (422)	0.000	0.13

### Negative Social Distance

Compared to the analysis of negative attitudes toward social distancing during COVID-19, a significant difference emerges between the averages obtained from the male participants in the research and those obtained from the male normative sample (An et al., 2021, see Table 8). The men of the normative sample referred to during the first pandemic period (An et al., 2021) scored higher than the males participating in the research. From the analysis of the results obtained by the participants in the research, no significant differences emerged related to gender, marital status, and educational qualifications. Instead, a significant difference emerged in relation to the age groups, particularly between those who were 18–24 years old (*M* = 2.48, SD = 0.78), 35–44 years old (*M* = 2.41, SD = 0.79), and 45–54 years old (*M* = 2.71, SD = 0.91) and those who are 55–64 years old (*M* = 1.93, SD = 0.68) and 65 or older (*M* = 1.84, SD = 0.58). Older people scored lower for negative social distancing than younger participants. Finally, a significant difference also emerged between those who were 25–34 years old (*M* = 2.22, SD = 0.73) and those who were 45–54 years old (*M* = 2.71, SD = 0.91); the latter had greater negative attitudes toward social distancing with respect to the former, *F*(5, 491) = 12.98, *p* < 0.01, $\eta_{p}^{2}$ = 0.12.

**Table 8:** Negative attitudes toward social distance: comparison between the average values of the participants and the average values of the Italian normative sample (An et al., 2021).

	Negative social distance		*t*(df)	*p*	Cohens’ *d*
	Participants	Italian normative sample during COVID-19			
	*M* (DS)	*M* (DS)			
Male	2.3 (1.0)	2.60 (1.13)	−2.534 (68)	0.014	0.28
Female	2.28 (0.75)	2.29 (1.00)	NS	0.821	

### Correlations and Regressions

From the analysis of the correlations reported in Table 9, it is clear how the fear of COVID-19 correlates positively with loneliness (*r* = 0.136, *p* < 0.01), the perception of mental illness (*r* = 0.178, *p* < 0.01), and a positive attitude toward social distancing (*r* = 0.161, *p* < 0.01). Loneliness correlates positively with the perception of mental illness (*r* = 0.433, *p* < 0.01), with a negative attitude toward social distancing (*r* = 0.184, *p* < 0.01).

**Table 9:** Correlations between the constructs investigated.

	1	2	3	4	5
1.UCLA_TOT	1	0.433^**^	−0.069	0.184^**^	0.136^**^
2.GHQ_TOT	0.433^**^	1	−0.063	0.074	0.178^**^
3.DIST_SOC_POS	−0.069	−0.063	1	−0.715^* *^	0.161^**^
4.DIST_SOC_NEG	0.184^**^	0.074	−0.715^**^	1	−0.063
5.FEAR OF COVID-19 SCALE	0.136^**^	0.178^**^	0.161^**^	−0.063	1

** *The correlation is significant at the 0.01 (two tailed) level*.

Further investigation highlighted the factors affecting the general health scale. The stepwise model selection in multiple linear regression analysis that considered the GHQ-12 scale as a dependent variable is presented in Table 10.

**Table 10:** Regression model: General health (GHQ-12) as dependent variable.

Variables^*^	B	SE	Beta	*t*	$R_{Adj}^{2}$
1.UCLA_TOT	0.810	0.079	0.417	10.27	
2.FEAR OF COVID-19 SCALE	0.492	0.164	0.122	3	0.199

* *In this model, the negative and positive social distance variables have been included but excluded from the model*.

The model has an *R*^2^ = 0.199, which means that 20% of the variance in the GHQ-12 scale is explained by the model. The *R*^2^ value was statistically significant. Loneliness (*β* = 0.122, *p* = 0.01) and fear of COVID-19 (*β* = 0.122, *p* = 0.003) were significant predictors.

## Discussion

From the analysis of the results, it emerges that in this particularly complex period, the perception of fear of COVID-19 affects the levels of psychological wellbeing of the population. Regarding the fear of COVID-19, the results show that compared to the first period of the pandemic, the participants in the research perceive lower levels of fear of COVID-19. This change may be because compared to the first wave, the government has implemented strategies to combat the spread of the virus, including the development of vaccines, which the majority of the population has received (Rania et al., in press); additionally, there has been a reduction in the rate of mortality, as reported by the National Institute of Statistics (Istat, 2021). It has also emerged that the perception of fear of COVID-19, albeit at lower levels than before, is an emotional state that has overwhelmed the population regardless of gender, age, marital status, current working modes, and educational qualifications. However, regarding perceived mental wellbeing, while the participants show a lower mental wellbeing compared to the normative sample, no significant differences emerge from the data collected during the first wave of the pandemic. Thee data are significant as it highlights how the malaise has significantly increased during the pandemic, as highlighted by several studies (Ahmed et al., 2020; Casagrande et al., 2020; Ferrucci et al., 2020; Moccia et al., 2020; Tian et al., 2020;Wang et al., 2020a,b; Yang and Ma, 2020; Rania and Coppola, 2021), and that it also remained high 1 year later despite the various strategies implemented to counter the spread of the virus. Furthermore, while there are no differences regarding the perception of mental health related to age group and gender. This result differs from what was found by La Rosa et al. (2021), who report how the women participating in their research reported a higher level of traumatic stress than men. Significant differences emerged in relation to marital status: a difference emerged between those who are single and those who are married. The latter reported lower levels of mental illness than the former. This may be because living with a partner and family in general can be considered a source of fundamental support, especially in situations where relational dynamics are experienced in a positive and satisfying way (Li and Wang, 2020; Parisi et al., 2021), particularly during moments of great complexity (Pyari et al., 2012). Finally, differences also emerged regarding the mode of working during COVID-19. Those who continued to work without changes in the mode reported a lower level of mental health than those who switched to smart working. This could be because the former felt less protected from a health perspective than those who were able to work from home; however, the most affected were those who lost their jobs during this emergency phase—they reported lower mental health scores. In fact, as highlighted in the literature, having a job has been described as a protective factor during the pandemic period (Li and Wang, 2020). Regarding the construct of loneliness, from the analysis of the results, it emerged that the perception of loneliness decreased significantly both when compared with the normative sample and when compared with data collected during the first wave of the pandemic. These results could be because following the imposed lockdown phase, the participants sought social activities in order to return to everyday life and cultivate their social relationships, which were significantly affected during the first phase of the pandemic, despite the availability of social networks.

Furthermore, from the results, it emerges that the youngest reported higher levels of loneliness, as also found by previous research (Li and Wang, 2020; Rumas et al., 2021), precisely because those most dedicated to activity were the most affected by the restrictions imposed. Finally, regarding marital status, the data show how single and divorced/separated were the most affected by loneliness; in fact, as also highlighted in the literature, the presence and support received from a family considerably influence the perceived level of loneliness (Rania and Coppola, 2021).

Regarding the positive attitude toward social distancing, the female participants reported a higher score than the women in the regulatory sample, showing a broader adherence to the restrictions imposed 1 year after their introduction into daily life. This result is in line with what emerged from the research conducted by An et al. (2021) and with the findings of a research conducted with a young population, which shows that women from a young age are more likely to adhere to the requests made by the authorities (Esposito et al., 2021).

Furthermore, contrary to what emerged from previous research (An et al., 2021), a general positive attitude toward social distancing emerges regardless of gender, marital status, current working modes, and educational qualifications. This attitude could be linked to the fact that the population has witnessed the deleterious effects of the pandemic, and this could have contributed to greater acceptance of the restrictions imposed. Finally, even with respect to this dimension, the elderly report more positive attitudes toward social distancing than the young, as also reported by previous research (An et al., 2021). This may be because the mortality rates caused by COVID-19 are higher among older people, who are more exposed to the risk of contracting the virus and its side effects (Onder et al., 2020). Regarding the negative attitude toward social distancing from the analysis of the results, it emerges that the men participating in the research obtained a lower score than the normative sample (from the first period of the pandemic). This could be because, over time, people have become accustomed to the imposed social distancing and have introjected this measure, perceiving it as a necessity to counter the spread of the virus. The results show that young people between 18 and 24 years old reported a higher score in this dimension than the elderly. This is probably because young people have been most socially affected by the health crisis, as founded by some research carried out in the era of COVID-19 (Cao et al., 2020; Li and Wang, 2020). Furthermore, as noted by Higuchi et al. (2020), staying at home has led to an excessive use of technologies in young people in particular. Furthermore, from the analysis of the correlations, there are positive correlations between the fear linked to COVID-19 and the perception of loneliness, mental health, and positive social distancing. In fact, the fear of COVID-19 leads to a favorable perception of social distancing to isolate oneself and consequently to perceive a low level of mental health. From the regression analysis, it emerges just how fear of COVID-19 and loneliness are predictors of perceived mental health, influencing people’s wellbeing. In this regard, Soraci et al. (2020) report that during other epidemics, some authors found associations between disorders, such as anxiety and depression and fear, which compromised the quality of life (Ford et al., 2018; Huang and Zhao, 2020a, 2020b), and note that these associations also occur in the current epidemic due toa social isolation, which had previously been shown to be strongly connected with anxiety and depression in both young and old individuals (Matthews et al., 2019; Santini et al., 2020).

## Conclusion

This study focused mainly on analyzing the fear of COVID-19 and social distancing and the repercussions on mental wellbeing and perceived loneliness by the participants. Although the study conducted represents an opportunity to illuminate the psychological consequences of the health crisis, there are limits that should be emphasized. The main limitation is due to the method of administration. While the online questionnaire made it possible to reach a larger number of participants, the lack of a predefined setting in which to dedicate themselves to completing it may have led the participants to provide careless answers (Ward et al., 2017). Furthermore, the use of the online questionnaire may have hindered the participation of some sections of the population less inclined to use technology. Moreover, although a large number of participants joined the research, it should be emphasized that there was an imbalance in participation in favor of women, as often happens in this type of research (Søgaard et al., 2004; Rania and Coppola, 2021); finally, while believing that this research helps to bring out the impact that COVID-19 has had on the mental health of the population, it should be emphasized that by not employing questions or exclusion criteria based on the presence of psychiatric or psychological comorbidities, it cannot be excluded that some participants may have previous psychological or psychiatric pathologies unknown to us. Despite these limitations, some strengths are represented by the fact that this research has made it possible to highlight how COVID-19 has led to nonnegligible psychological consequences even 1 year after the most critical phase; moreover, the large number of participants, who joined voluntarily and without any type of reward, made it possible to determine differences related to some sociodemographic variables analyzed, including age, marital status, and work modes. To conclude, the results emerging from this research should make policymakers reflect on the need to find containment strategies and tools for this pandemic or other health crises that have a limited impact on the sociopsychological wellbeing of the population.

## Data Availability Statement

The datasets presented in this article are not readily available because the datasets generated for this study cannot be shared for ethical reasons related to privacy; however, the authors will attempt to make the data available for valid requests. Requests to access the datasets should be directed to NR, nadia.rania@unige.it.

## Ethics Statement

Ethical review and approval was not required for the current study in accordance with the local legislation and institutional requirements. Research was carried out in accordance with the Ethics Research Recommendations of the American Psychological Association (APA) and in accordance with the Declaration of Helsinki. Participation was entirely voluntary, confidential and anonymous. The participants were informed that they were free to withdraw from the study at any time. The patients/participants provided their written informed consent to participate in this study.

## Author Contributions

NR: conceptualization, visualization, supervision, and project administration. NR and IC: methodology, formal analysis, investigation, data curation, writing—original draft preparation, and writing—review and editing. All authors have read and agreed to the published version of the manuscript.

## Conflict of Interest

The authors declare that the research was conducted in the absence of any commercial or financial relationships that could be construed as a potential conflict of interest.

## Publisher’s Note

All claims expressed in this article are solely those of the authors and do not necessarily represent those of their affiliated organizations, or those of the publisher, the editors and the reviewers. Any product that may be evaluated in this article, or claim that may be made by its manufacturer, is not guaranteed or endorsed by the publisher.

## Footnotes

^1^ https://opendatadpc.maps.arcgis.com/apps/dashboards/b0c68bce2cce478eaac82fe38d4138b1
